# Male Fern in Tape Worm

**Published:** 1864-05

**Authors:** 


### MALE FERN IN TAPE WORM.

Dr. Alexander Fleming gives the following as his result of his extensive therapeutical inquiries as to the usefulness or otherwise of the oil of male fern in tapeworm, and the best mode of exhibiting the drug. These inquiries embrace 100 cases.

*Sex.* Of these 100 cases, 30 were males, and 70 females.

The remarkable preponderance of the female sex among the subjects of tapeworm, here shown, and, as I believe, for the first time on numerical data, is full of interest in relation to the cause of the disease, and most deserving of further inquiry. The great majority of the cases embraced in this report are taken from hospital out-patients, among whom the women suffer frequently from dyspepsia, very much more so than do the men; and we can readily understand how the "measle" will have a higher chance of escaping death in a weak stomach, and subsequently making a home for itself in the bowels. As respects the diet itself, the risk run by men must be greater than that by women; as they eat a larger proportion of animal food, and, in Birmingham especially, of pork.

Our returns show that the male fern, as a remedy, is of equal efficacy in both sexes.

*Age.* The age of the patient is not mentioned in 8 of the cases. Of the remaining 92, the average age of all, in round numbers, is 29; of the females, 30; of the males, 28. The returns include cases of all ages except infancy, and prove that the oil of male fern is an efficient remedy as well in the child as in the adult. A child of 1 year and 11 months is the youngest, and a woman aged 69 the oldest example. The exclusive milk diet of infants, and consequent freedom from the cause of the parasite, explains their immunity from tapeworm.

*The Duration of the Disease* is not given in 33 cases. Of the remaining 67, it is stated to vary from a few days, as in 4 cases in Dr. Anderson's schedule, to 36 years, as in the example reported by Mr. Anderton. There are 11 cases whose duration varies from 6 weeks to 10 months; 16 are reported of one year's duration; 9 of 2 years; 4 of 5 years; 3 of 7 years; 3 of 10 years; 1 of 12 years; 1 of 14 years; 2 of 20 years; and 1 of 36 years. The returns show that the oil of male fern has been as efficient as a remedy in cases of long standing as in the more recent.

*Previous Treatment.* In 35 of the cases, it is stated that there was no previous treatment. Among the remedies which had been used in the others, kousso was employed twice—once with, and once without success. Turpentine had been given on fifteen occasions—seven times with, and eight times without success. The oil of male fern had been previously used five times—three times with, and twice without success. In one of those cases where it had failed, it was subsequently given in mixture with milk, in the mode which I have suggested, and with perfect success.

*Dose, Time, and Mode of Administration. Dose.* The medicine has been administered in doses of a few minims, of half a drachm, of one drachm, one and a half, and of two drachms. The returns show that one drachm is a sufficient dose; at least, in the great majority of cases. The larger doses more frequently excite sickness, vomiting and diarrhœa.

*Time.* In many of the cases, the oil was given in the morning; in a greater number, at bedtime. The results of the two methods, when compared together, do not show any material difference in success. I prefer to give the drug at bedtime, because the patient should continue to fast for eight or ten hours after taking it; and it is easier to do so during sleep than waking.

*Mode.* In 47 of the cases, the oil was given with milk, in the manner which I had myself suggested in the observations which accompanied the schedule. The following is the formula referred to:

"Mix well of oil of male fern one drachm, and mucilage half an ounce. The draught is mixed with one ounce and a half of sweet milk, and taken at bedtime; the patient having omitted the dinner and evening meal of that day. Taken thus, on an empty stomach, the mixture is speedily carried into the intestines, to feed, and at the same time poison, the hungry parasite which nestles there. Milk is the favorite food of the worm. Next morning, a dose of castor oil may be given. If necessary, this medication may be repeated daily, one, two, and three times, or until the worm is discharged."

In the remaining cases, the drug was given without milk, in mucilage or some aromatic water. In nearly all these cases comprised in the returns, care was taken to give the remedy on an empty stomach. The two classes of cases, therefore, or those in which the male fern was given with milk, and those in which milk was not used, admit of a fair comparison; and of the higher efficiency of the first of these methods of exhibition the returns are conclusive. So given, the drug acts more quickly, and at the same time more efficiently. The proportion of failures is nearly the same with both methods; but the length of worm discharged, and, so far as we can judge, the thoroughness of the cure, predominate in those cases where milk was used.

*Physiological Effects.* Sometimes the medicine operates without pain or nausea; more often, there are sickness, griping pains, and purging. Vomiting is reported in ten of the cases. Dr. Bree observes that under its use, the urine was usually loaded with lithic acid. In one of Dr. Anderson's cases, the menses, which had been absent for several months, returned after the use of the oil. The vomiting and purging were caused frequently by the second dose, after the worm had been discharged; and must be ascribed to the action of the drug itself on the gastro-intestinal mucous membrane—not, as some have thought, to the dying struggles of the poisoned worm, though it may be that these play some part in their causation.

In five of the 100 cases, the worm was discharged alive. Except that it was expelled with unusual speed, I cannot trace any circumstances to account for the living state of the parasite in these examples.

The largest portion of tapeworm which is reported to have been passed, is fifteen yards. This was in Dr. Bennett's case. No mention is made of any other species of tapeworm than the tænia solium. Large round worms were discharged in two cases.

The worm was, for the most part, expelled after the first dose, but in a few cases not till after the second or third dose. The worm was often passed before any purgative was taken, and separately from the ordinary evacuation. In one instance ecorded in Mr. Thompson's schedule, the worm was discharged upwards by vomiting. This was the case of a female aged 40, who had suffered many years from tapeworm. She took one drachm of the oil of male fern in milk, according to my formula; and, in the course of an hour, vomited a very long tapeworm, which was quite dead. None passed by stool. After two days the draught was repeated, and she passed a large quantity of dead and broken tapeworm. The patient had previously taken various remedies without success. In Dr. Anderson's schedule, the case of a girl aged 18 is narrated, who became very sick after taking two drachms of the oil of male fern in milk, and vomited a large round worm. She was afterwards purged smartly, and passed a quantity of joints of tapeworm.

The average time which elapsed between the administration of the oil and the expulsion of the parasite was six hours. It was discharged in half an hour in seven cases, in one hour in nine cases, in two hours in six case, in three hours in three cases, in five hours in six cases. The longest interval mentioned is twenty-four hours.

In several of the cases the worm was passed in a broken and softened state. In these cases, a considerable interval had elapsed between the taking of the oil and the expulsion of the worm, the softened condition of which was probably due to a more or less complete digestion of the already poisoned and dead worm.

The head is reported to have been found in three cases (schedule of Mr. Spencer); but, in one of these, its discovery rests only on the authority of the patient. It is generally thought that the rarity with which the head is obtained is due to its not being killed and detached with the body; but it seems improbable that the poison should take more effect on the body than the head of the creature, and which it meets first in its passage downwards from the stomach. According to Dr. Nelson, the food is taken in chiefly by the head. I am more inclined to refer the rare discovery of the head to its solution in the digestive fluids. Thin and delicate, it must be easy of digestion. Moreover, placed higher up in the canal, it is in closer proximity to the more active solvent juices. The thin and translucent neck, though found more often than the head, is also generally absent; and probably for the like reason. I am disposed to refer relapses to the growth of other worms, which have escaped the poison, and not to the resprouting of the old head.

*Duration of the Cure.* Though relapses often occur, there is reason to believe that the cure is permanent in a large proportion of the cases. The length of time (one year) assigned to his inquiry, and the difficulty of ascertaining the future history especially of hospital patients, render the returns in reference to this important point unavoidably of less value than we could desire. I may mention in this place, that Mr. Osborn in a note to his schedule, states that two cases of tapeworm are known to him, both females, of 38 and 17 years of age respectively, where the oil of male fern was used with success, and where the patients remained, to his knowledge, well for many years.

In concluding this report, it is only just to remember, in connection with our subject, the early labors of Peschier, of Geneva, and dating so far back as 1830, but which ha l been almost overlooked in England until Dr. Christison, in 1853, gave the sanction of his authority to the results of Peschier's trials. The later experiences of Drs. Gull, Jenner, Bennett, Willshire, Ransome, and others, have abundantly confirmed their observations, and, conjoined with the results of the present inquiry, establish beyond doubt the great efficacy of oil of male fern in tapeworm, and its superiority to the other known remedies of this disease. Further, our report points very decidedly to the most efficient mode of exhibiting the drug; and the whole inquiry has, as I have reason to know, rendered excellent service to therapeutics by making the virtues ot the oil of male fern more widely known and employed through the profession.

It remains only for me to offer my best thanks to all the gentlemen who made returns to me, for their valuable aid in this inquiry.—*British Med. Journal*, Jan. 16, 1864.

